# Enhanced Innate and Acquired Immune Responses in Systemic Sclerosis Primary Peripheral Blood Mononuclear Cells (PBMCs)

**DOI:** 10.3390/ijms241914438

**Published:** 2023-09-22

**Authors:** Iulia Szabo, Medeea Badii, Ildikó O. Gaál, Robert Szabo, Radu A. Popp, Leo A. B. Joosten, Tania O. Crişan, Simona Rednic

**Affiliations:** 1 Department of Rheumatology, “Iuliu Hațieganu” University of Medicine and Pharmacy, 400012 Cluj-Napoca, Romania; rotaru.iulia@umfcluj.ro (I.S.);; 2 Department of Rheumatology, County Emergency Hospital, 400347 Cluj-Napoca, Romania; 3 Department of Medical Genetics, “Iuliu Hațieganu” University of Medicine and Pharmacy, 400012 Cluj-Napoca, Romania; 4 Department of Internal Medicine, Radboud Institute for Molecular Life Sciences (RIMLS), Radboud University Medical Center, 6525 GA Nijmegen, The Netherlands; 5 2nd Anesthesia Department, “Iuliu Hatieganu” University of Medicine and Pharmacy, 400012 Cluj-Napoca, Romania; 6 Department of Anesthesia and Intensive Care, County Emergency Hospital, 400347 Cluj-Napoca, Romania

**Keywords:** systemic sclerosis, cytokines, PBMCs, innate

## Abstract

Chronic immune activation in systemic sclerosis is supported by the production of a plethora of cytokines with proven regulatory activities of the immune responses. This study aimed to explore PBMCs’ cytokine profiles in SSc patients versus controls, as well as to investigate the balance between pro- and anti-inflammatory cytokines in association with disease duration. PBMCs were isolated from 18 SSc patients and 17 controls and further subjected to in vitro stimulation with lipopolysaccharide and heat-killed *Candida albicans*. Cytokine production was measured after 24 h and 7 days, respectively, using ELISA kits for interleukin (IL)-1β, IL-1 receptor antagonist (IL-1Ra), IL-6, tumor necrosis factor (TNF), IL-10, IL-17, and interferon-gamma (IFN-gamma). IL-1 β, IL-6, and TNF levels were increased in SSc patients compared with healthy volunteers irrespective of the stimulus used. IL-1Ra and Il-17 concentrations were not statistically different between groups, even though a trend toward higher levels in patients compared with their matched controls was also observed. Most cytokines demonstrated a stable course with disease progression, except for IL-10 levels, which declined over time. In conclusion, the results of this pilot study reveal that in patients with SSc a persistently enhanced immune response is established and maintained regardless of stimulus or disease duration.

## 1. Introduction

Systemic sclerosis (SSc) is a chronic connective tissue disease characterized by a distinct constellation of pathological pathways that lead to inflammation, functional and structural microvascular alterations, and progressing fibrosis affecting the skin and internal organs [1]. Immune dysregulation of both innate and adaptive responses in SSc is supported by various lines of evidence showing mononuclear cell tissue infiltration, sustained production of pro-inflammatory cytokines through persistent activation of Toll-like receptors (TLRs) by damage-associated molecular patterns (DAMPs), the activation of plasmacytoid dendritic cells (pDCs), and increased expression of type I interferons (IFN), along with the activation of T and B lymphocytes in skin and peripheral blood [2,3,4]. The expression profiles of cytokines and their known interactions provide insight into the role of the immune system in SSc pathogenesis and might as well reveal new therapeutic targets [5]. In a previous report, the co-culture of activated SSc macrophages releasing CCL2, IL-6, and TGF-β with SSc fibroblasts induced fibroblast activation, thus highlighting the involvement of both innate immune cells and cytokines in modulating fibrogenesis [6]. The relationship between cell-mediated immunity and end-organ disease was also demonstrated by *Brown* et al. in both children and adults suffering from diffuse cutaneous SSc (dcSSc) [7]. Peripheral blood mononuclear cells (PBMCs) isolated from these patients and stimulated in vitro with solubilized type I collagen (CI) or constituent α1 (II) and α2 (I) polypeptide chains exhibited enhanced concentrations of platelet-derived growth factor (PDGF)-AA, PDGF-BB, TNF-α, IL-13, and EGF. Subsequent exposure of dcSSc dermal fibroblasts to harvested supernatants resulted in decreased production of matrix metalloproteinase-1 (MMP-1) [7]. Targeting the immune responses can be a promising therapeutic approach, as recently shown in the work by de Almeida et al. [8]. Isolated PBMCs from patients with SSc were subjected to stimulation with phytohemagglutinin (PHA) and treatment with LPSF/GQ-16, a thiazolidinediones derivative. Concomitantly, intraperitoneal injections of LPSF/GQ-16 were administered to a hypochlorous acid (HOCl)-induced mouse model of SSc. Results showed that LPSF/GQ-16 not only influenced the PBMCs’ production of IL-2, IL-4, IL-6, IL-17A, TNF, and IFN-γ, but also reduced skin thickening, downregulated biomarkers of skin and lung fibrosis, as well as decreased the activation of immune cells in the experimental model [8].

The short half-life of cytokines and their interaction with cytokine-binding proteins, serum inhibitors, and soluble cytokine receptors influence their circulating levels [9]. Therefore, stimulated production of cytokines by PBMCs could offer more insight into the role and reactivity of the immune response as opposed to serum levels [10]. Prior studies have generated some conflicting results concerning PBMCs’ cytokine signatures in SSc patients, further supporting the need for more research in this area [11,12,13].

The main objective of this pilot study was to assess various cytokine profiles in primary PBMCs upon stimulation with lipopolysaccharide (LPS) and heat-killed *Candida albicans*, as well as their expression levels in correlation to disease duration. Moreover, we explored the potential associations between serum cytokine concentrations and different clinical features.

## 2. Results

### 2.1. Basic Clinical Parameters of Participants

A detailed description of the patients’ characteristics is provided in Table 1. All 18 patients with SSc and 17 healthy controls (HC) were age-matched females. The median (IQR) age in the SSc cohort was 50 (45, 56) years, whereas that in the control cohort was 48 (44, 53) years. According to LeRoy’s classification, 50% had diffuse cutaneous SSc, 44% limited cutaneous SSc, and 6% SSc sine scleroderma with a median (IQR) modified Rodnan skin score (mRSS) of 5.5 (4.0, 8.8) [14]. The median (IQR) disease duration (from disease diagnosis) was 4.0 (2.2, 9.2) years and seven (39%) patients had early disease (≤3 years from diagnosis). Endoscopy revealed reflux esophagitis in 6 (40%) patients, while interstitial lung disease (ILD) as well as a previous or current history of digital ulcers was present in 11 (61%) and 10 (56%) patients. None of the recruited patients experienced scleroderma renal crisis, conduction blocks, or pulmonary arterial hypertension (PAH). Other severe internal organ involvement such as arrhythmias requiring therapy or musculoskeletal manifestations was not prevalent in our study cohort. Regarding treatment, none of the patients had been treated with glucocorticoids at the time of recruitment, one patient did not require any immunomodulatory therapy at all, two patients were prescribed biological drugs (tocilizumab), while the remainder had received immunosuppressive or conventional synthetic antirheumatic drugs either alone or in combination.

### 2.2. Patients with SSc Show Elevated Ex Vivo Cytokine Production Capacity

**Figure 1:**
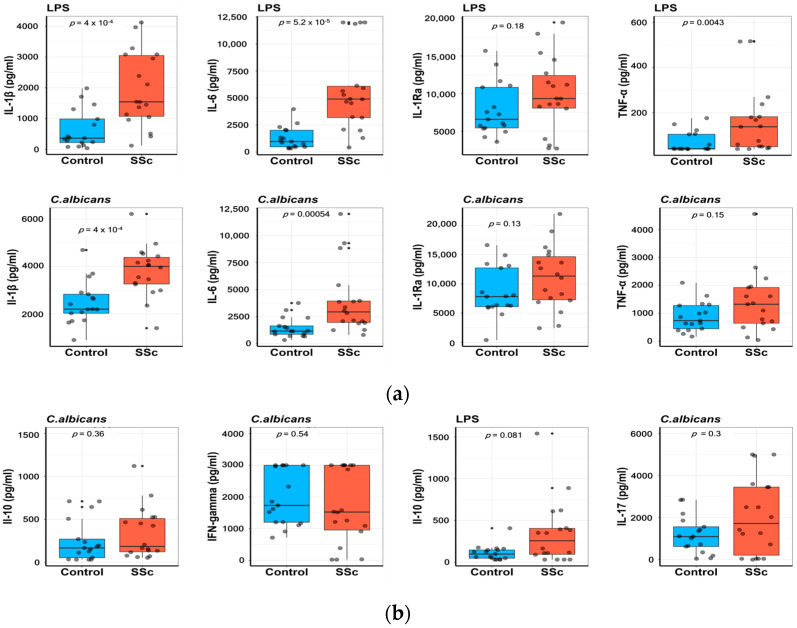
Enhanced cytokine response in PBMCs from patients with systemic sclerosis (SSc) compared with healthy controls (HC). Isolated PBMCs were stimulated in vitro for 24 h and 7 days with LPS and heat-killed Candida albicans. Cytokine levels were measured in the supernatants using commercial ELISA kits for interleukin (IL)-1β, IL-1Ra, TNF-α, and IL-6 (**a**), as well as IL-17, IFN-gamma, and IL-10 (**b**). Data are shown as median (IQR), Wilcoxon rank-sum test. Each dot represents a sample.

Freshly isolated PBMCs from patients with SSc and HC were incubated for 24 h with LPS and heat-killed Candida albicans. IL-1 β and IL-6 levels were significantly increased in SSc patients compared with HC after exposure to either LPS or Candida albicans. TNF production was enhanced irrespective of the stimulus used, but only reached statistical significance after LPS exposure. IL-1 receptor antagonist (IL-1Ra) elevations were not statistically significant between groups. (Figure 1a).

Stimulation of PBMCs for 7 days with Candida albicans and LPS in the presence of 10% human pooled serum was followed by measurements of IL-17, IFN-gamma, and IL-10 levels. A trend toward higher concentrations of IL-10 was observed in SSc patients compared with their matched controls, with a *p*-value of 0.081. Moreover, no difference was found in the production of Il-17 and IFN-gamma between groups (Figure 1b).

### 2.3. Assessment of Cytokine Production in Correlation to Disease Duration

**Figure 2:**
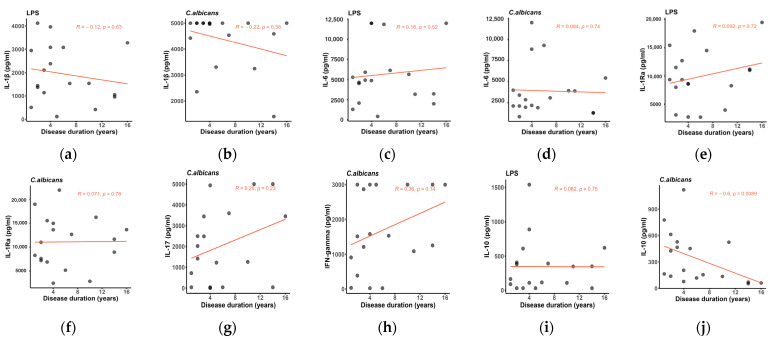
(**a**–**j**) Scatter plots showing the correlation analysis between induced cytokine production in PBMCs from SSc patients and disease duration, Spearman’s correlation coefficient. The fitted linear regression line of each scatter plot (red) is depicted along with the R and *p*-values. IL-17 and IFN-gamma registered a positive trend with disease progression, while IL-10 concentrations significantly decreased with increasing disease duration. This correlation was evident only upon stimulation with heat-killed Candida albicans.

Inflammatory parameters of patients with SSc are typically expected to diminish in time, as fibrotic mechanisms are established [15]. We assessed the cytokine production in PBMCs of patients with SSc in association with disease duration. Interestingly, as shown in Figure 2, cytokine levels tend to maintain a stable course as the disease progresses, except for IL-10 concentrations, which decline over time (r = −0.6, *p* = 0.0089).

### 2.4. Cytokine Production in Relationship to SSc Subphenotypes

**Figure 3:**
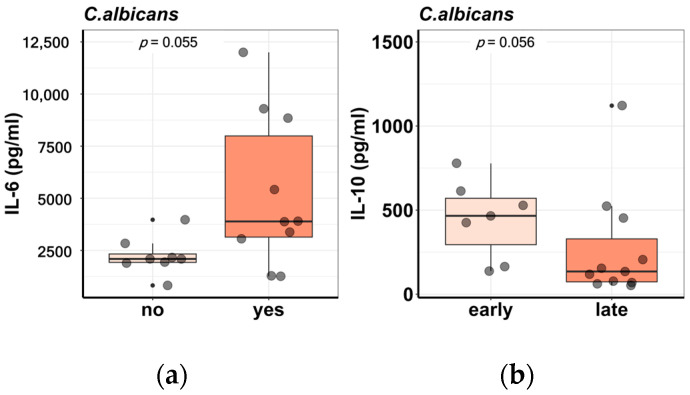
Differential cytokine production in PBMCs from SSc patients in relation to disease phenotypes. (**a**) IL-6 levels are higher in patients with a current or previous history of DUs as opposed to patients who never experienced this disease complication. (**b**) IL-10 concentrations are higher in patients with early disease compared with patients with late disease. Each dot represents a sample.

Additionally, the differential cytokine production in PBMCs from SSc patients was analyzed in relation to several disease characteristics. The induced responses in PBMCs were similar between disease subsets, diffuse cutaneous (dc) versus limited cutaneous (lc) SSc. The presence of ILD on high-resolution computed tomography (HRCT) scans as well as a previous or current history of digital ulcers (DUs) were not associated with a differential cytokine profile, apart from IL-6 reaching a borderline statistical significance for higher induced levels in patients with DUs upon stimulation with heat-killed Candida albicans (*p* = 0.055) (Figure 3a). Moreover, autoantibody subtypes were not associated with the production of innate immune cytokines (IL-1 β, IL-6, IL-1Ra) or T helper response (IL-17, IFN-gamma) after in vitro stimulation of PBMCs. No correlation could be found between cytokine levels and mRSS, but early disease was characterized by increased levels of IL-10 (Figure 3b). Other disease phenotypes, such as cardiac involvement, pulmonary hypertension, renal crisis, and arthritis, have been taken into consideration, but were not included in the analysis because of either low prevalence or missing data.

## 3. Discussion

The role of inflammation and immune dysregulation in the pathogenesis of SSc is a well-known phenomenon. Even from the early stages of the disease, mononuclear cells, macrophages, T and B lymphocytes, mast cells, and natural killer cells infiltrate tissues and accumulate in a perivascular distribution [16]. These cells produce a plethora of cytokines that exert proinflammatory and profibrotic effects, leading to cell recruitment and the production of extracellular matrix components [12]. T cell activation, Th2 polarization resulting in a Th1/Th2 cytokine imbalance, as well as evidence of an increased expression of type I IFN-inducible genes in peripheral blood lymphocytes further support this hypothesis [17,18,19,20].

Our data highlight the ability of PBMCs to mount an enhanced immune response in SSc patients compared with their matched controls in response to LPS or heat-killed Candida albicans. The increased cytokine production is particularly evident for IL-1 β and IL-6 (Figure 1a), while other cytokines (IL-1Ra, TNF, IL-17, IL-10) exhibit higher concentrations in patients versus controls but without reaching statistical significance (Figure 1a,b). IFN-gamma production showed no difference between groups (Figure 1b). On the one hand, these results are in line with the published literature. Umehara et al. showed that upon stimulation with LPS, purified monocytes isolated from SSc patients produced increased concentrations of TNF [13]. Phytohemagglutinin (PHA)-stimulated PBMCs released higher levels of IL-6, IL-10, IL-18, macrophage inflammatory protein (MIP)-1α, and TNF [21]. Enhanced concentrations of IL-4 and IFN-gamma were measured in supernatants of PBMCs from SSc patients after in vitro cytomegalovirus (CMV) antigen stimulation [22]. On the other hand, Kantor TV et al. demonstrated decreased mean values of IFN-gamma after PBMC incubation with PHA, while in vitro LPS-induced production of TNF and IL-1β was comparable between patients and normal PBMCs [11]. In another study, anti-CD3/CD28 PBMC stimulation resulted in lower levels of TNF, Il-10, and IL-2 in SSc compared with controls [12].

Remarkably, in our study, no distinct cytokine signatures could distinguish between different disease phenotypes (Figure 3). However, IL-6 levels proved to be higher in patients with a previous or current history of digital ulcers, with a borderline *p*-value of 0.055. Likewise, patients with early disease (≤3 years from diagnosis) produced higher concentrations of IL-10 but without crossing the threshold for statistical significance (*p* = 0.056). Dantas et al. demonstrated that patients with lung fibrosis exhibited increased levels of IL-2 and IL-4, whereas patients with digital ulcers displayed higher concentrations of IL-10 and IL-4 after anti-CD3/CD28 PBMC stimulation [12]. However, most of the studies focused on the detection of serum rather than PBMC spontaneous or -induced cytokine responses and their respective clinical associations. Consequently, positive correlations were found between higher serum IL-6 concentrations and disease duration, European Scleroderma Study Group activity score, HRCT-ground glass score, and HRCT-fibrosis score, while negative correlations were described for DLCO% and 6 min walk distance [23]. Similar results were found by Lin et al., who proved that IL-6 levels were higher in patients with disease duration ≥ 5 years, interstitial lung disease, pulmonary arterial hypertension, and gastrointestinal and cardiac involvement [24]. Another group described positive correlations between serum levels of IL-6 and IL-10 and mRSS, together with a positive relation between IL-10 and pulmonary fibrosis [25]. A different study involving a large cross-sectional cohort of SSc patients and healthy donors also underlined the link between interstitial lung disease and elevated serum IL-6 [15]. In addition to SSc, the relationship between IL-6 and lung fibrosis was observed in other diseases as well, such as COVID-19-induced respiratory failure [26]. However, this association could not be validated when the number of patients was small [27]. Overall, the limited number of participants enrolled in our study together with missing baseline characteristics hindered our ability to establish relevant differences in cytokine production according to distinct disease parameters. Nevertheless, this major limitation can be successfully overcome with larger cohorts [28,29].

Notably, most cytokine levels remain persistently elevated in our group despite disease progression (Figure 2). The only significant correlation identified was between decreasing levels of IL-10 and increasing disease duration (Figure 2l). In addition to its anti-inflammatory activity, IL-10 modulates fibroblast function and collagen production via the TGF-β/Smad2 signaling pathway in alternatively activated (M2) macrophages [30,31,32]. Accordingly, the study by Laurent et al. confirmed that TGF-β dramatically depressed in vitro *KLRG1* expression on innate lymphoid cells (ILC) 2 and induced profibrotic responses in dermal fibroblasts through IL-10 downregulation [33]. These observations suggest that decreased IL-10 expression might play a role in the development of a more fibrotic phenotype as the disease progresses. Additionally, previous articles revealed the involvement of Th17 cells in the pathogenesis of SSc [34]. A recent paper showed that the frequency of IFN-γ^+^IL-17^+^Th17 cells isolated from peripheral blood samples was increased in SSc compared with controls and correlated with enhanced mRNA levels of IFN-γ and IL-17. Moreover, IFN-γ^+^IL-17^+^Th17 cells were associated with increased disease duration, disease activity, and mRSS. Co-culture of IFN-γ^+^IL-17^+^Th17 cells with dermal fibroblasts led to the upregulation of α-SMA and COL1A1. Surprisingly, pretreatment with the neutralizing anti-IL-21 antibody of IFN-γ^+^IL-17^+^Th17 cells before incubation with dermal fibroblasts resulted in the downregulation of α-SMA and COL1A1 mRNA, suggesting a contribution from IL-21 in SSc fibrogenesis [34].

The ability of stimulated PBMCs to elicit a persistently increased production of cytokines in SSc patients compared with controls enables us to hypothesize about a potential role for innate immune memory (trained immunity) in the pathogenesis of this disease. Trained immunity refers to the ability of innate immune cells previously exposed to certain infectious or endogenous antigens to mount an enhanced response upon restimulation. This process is established and maintained through complex metabolic and epigenetic reprogramming of peripheral and bone marrow myeloid precursors [35,36,37,38]. In SSc, trained immunity can both accelerate or inhibit inflammatory and fibrotic processes depending on the initial stimulus [39]. In a mouse model of SSc-, BCG- and low-dose LPS-trained macrophages exhibited different cytokine signatures and epigenetic rewiring: low-dose LPS downregulated inflammation and fibrosis, while BCG stimulation exerted an opposite effect. Furthermore, peritoneal injections of low-dose LPS led to decreased collagen deposition in the skin and lungs of mice as well as downregulated mRNA expression levels of α-SMA, COL1A1, and TGFβ-1. Collectively, these results highlight the potential role of cellular therapy in shaping immune responses in SSc [40]. Likewise, IFNβ stimulation of mouse embryonic fibroblasts and bone marrow-derived macrophages generated increased levels of various IFNβ-stimulated genes in pretreated cells compared with naive cells, granting transcriptional memory through the recruitment of RNA polymerase II and acquisition of epigenetic marks, primarily H3.3 and H3K36 trimethylation. Given the type I IFN signature in SSc, a similarly acquired epigenetic memory could also contribute to disease pathogenesis [41]. Epigenetic reprogramming requires increased energy metabolism, which is achieved in trained innate immune cells through a shift from oxidative phosphorylation and fatty acid oxidation to aerobic glycolysis [42,43]. RNA sequencing and genome-wide genotyping of monocyte-derived macrophages was performed in SSc patients and revealed an upregulation in the expression levels of genes involved in various metabolic pathways, including glycolysis, hypoxia, and mTOR signaling [44], further suggesting that trained immunity-like processes may play a role in the persistent deregulation of immune responses in SSc.

In SSc, specific PBMC subsets are differently abundant in the intravascular versus skin compartment, suggesting an adaptation of the immune cells in response to the environmental milieu [45]. Recently, Gur et al. [45] performed single-cell genomic analysis of immune and stromal cells in a heterogeneous cohort of 97 SSc patients and 56 healthy controls, generating a map of eight large immune cell lineages, with various subsets of dendritic and NK cells, Langerhans cells, skin effector and regulatory T cells, macrophages, as well as mast cells being preferentially expressed in the skin and distinct from the immune subsets found in the blood. Surprisingly, further differential composition analysis could not identify a difference in immune cell subsets in the skin or blood of SSc patients compared with healthy subjects. After accounting for disease characteristics, subsequent sub-analysis revealed discrete immune signatures, particularly in the blood and skin of diffuse cutaneous SSc patients with early or active disease. A reduction in the number of naive T cells was observed in the blood of both SSc subsets, accompanied by an increase in classical and non-classical (CD16+) monocytes as well as the upregulation of dendritic and plasma cells in the diffuse cutaneous subtype. Furthermore, an association between anti-Scl-70 antibodies and higher levels of blood monocytes was also highlighted [45]. Taken together, the work by Gur et al. indicates that patients with SSc exhibit distinct immune cell perturbations in the peripheral blood compartment. These immune dysregulations could potentially contribute to the enhanced cytokine production observed in our cohort, suggesting a new direction for research that could facilitate our understanding of SSc pathogenesis.

Our study has several limitations. Sampling bias could have resulted from the fact that these are patients from a tertiary care hospital, and therefore the group might not truly reflect the entire spectrum of the disease. Moreover, the relatively mild SSc phenotypes of the patients included in our work and the gender bias could have further contributed to the underrepresentation of the SSc cohort. The restricted sample size together with missing clinical and laboratory data limited our ability to identify specific disease subtypes according to their cytokine expression profiles. This outcome might have also been influenced by the limited number of stimuli used and the specific measured cytokines. Additionally, the cross-sectional design of this study does not reflect the dynamic changes in cytokine profiles during long-term follow-up. Lastly, we did not concomitantly detect the serum concentrations of the cytokines of interest, and therefore we cannot corroborate the induced responses in PBMCs with the circulating levels of the investigated analytes.

## 4. Materials and Methods

### 4.1. Study Design, Participants, and Variables

Eighteen consecutive SSc patients attending the Rheumatology Department at the tertiary care County Emergency Hospital Cluj-Napoca, “Iuliu Hațieganu” University of Medicine and Pharmacy, Cluj-Napoca, were prospectively enrolled in a cross-sectional study between February 2020 and October 2021. All participants were aged 18 years or older and fulfilled the 2013 American College of Rheumatology/European Alliance of Associations for Rheumatology (ACR/EULAR) Classification Criteria for SSc. Patients with overlapping syndromes were excluded from the study. Demographic, clinical, and laboratory parameters were retrieved from medical charts. The extent of skin involvement was assessed with a modified Rodnan skin score (mRSS) and patients were further subclassified into limited cutaneous, diffuse cutaneous, or sine scleroderma (limited SSc) following LeRoy et al.’s criteria [14]. Interstitial lung disease (ILD) was defined as the presence of ground-glass opacification, reticular changes, traction bronchiectasis, or honeycombing in a bibasilar distribution on high-resolution computed tomography (HRCT) scans. Pulmonary arterial hypertension (PAH) screening and referral for right heart catheterization (RHC) was performed in agreement with the 2015 European Society of Cardiology/European Respiratory Society guidelines [46]. Esophageal involvement was evaluated via endoscopy. Conduction blocks and arrhythmias requiring therapy were considered disease-related cardiac manifestations. Scleroderma renal crisis was described as an abrupt onset of severe hypertension accompanied by acute kidney injury [47]. Autoantibody status, routine laboratory parameters, pulmonary function tests, and echocardiographic measurements of left ventricular systolic function were collected from patient records. Time since diagnosis to blood sample collection was established as disease duration, with patients being categorized as having either early disease (≤3 years) or late disease (>3 years). Seventeen age- and gender-matched controls with no history of a systemic autoimmune disease were recruited from the hospital personnel. All participants gave written informed consent before being included in the study. Approval for the current study was granted by the Ethics Committee of „Iuliu Hațieganu” University of Medicine and Pharmacy, Cluj-Napoca (approval no. 104/09.03.2020). This study was conducted in accordance with the Declaration of Helsinki.

### 4.2. Cell Preparation and Culture Conditions

Peripheral blood was collected from the cubital vein of patients with SSc and healthy volunteers into 10 mL EDTA tubes under sterile conditions. PBMCs were separated using Ficoll-Paque (GE Healthcare, Chicago, IL, USA) density gradient centrifugation and washed 3 times with phosphate-buffered saline (PBS) at 4 °C. Cells were counted using the Coulter Counter method and the number was adjusted to 10 × 10^6^ PBMCs/mL in Dutch Modified Roswell Park Memorial Institute (RPMI) 1640 medium (Sigma, St. Louis, MO, USA). Cells were cultured in 96-well plates at 5 × 10^5^ PBMCs in a volume of 50 µL per well with Dutch Modified RPMI 1640 medium (Sigma, St. Louis, MO, USA) supplemented with 50 µg/mL gentamycin (Sigma), 2 mM GlutaMAX (Gibco, Grand Island, NY, USA), and 1 mM pyruvate (Gibco, Grand Island, NY, USA).

PBMCs were stimulated for 24h in a round-bottom plate or 7 days in a flat-bottom plate with lipopolysaccharide (LPS) (0.1 µg/mL) and heat-killed Candida albicans (10^6^ col/mL). Day 7 experiments were supplemented with 10% human pooled serum. Each experiment was performed in replicate wells and supernatants were pooled before measurement and stored at −20 °C until cytokine measurements were conducted.

### 4.3. Cytokine Measurements

Supernatants collected at 24 h and 7 days were used for cytokine measurements using enzyme-linked immunosorbent assay with the respective sandwich ELISA kits for IL-1β, IL-1Ra, IL-6, TNF (24 h), IL-10, IL-17, and IFN-gamma (7 d) following the manufacturer’s instructions (R&D Systems, Minneapolis, MN, USA). The absorbance at 450 nm was measured with Synergy HTX Multi mode reader from Bio-Teck. The lowest range of detection was 39 pg/mL for IL-1β, 390 pg/mL for IL-1Ra, 39 pg/mL for TNF, 94 pg/mL for IL-6, 9 pg/mL for IFN-gamma, 16 pg/mL for IL-17, and 31 pg/mL for IL-10. Samples were diluted prior to assay, 10-fold for IL-1β and IL-1Ra, 20-fold for IL-6, 5-fold for TNF, 2-fold for IFN-gamma, 2-fold for IL-17, and 2-fold for IL-10.

**Figure 4:**
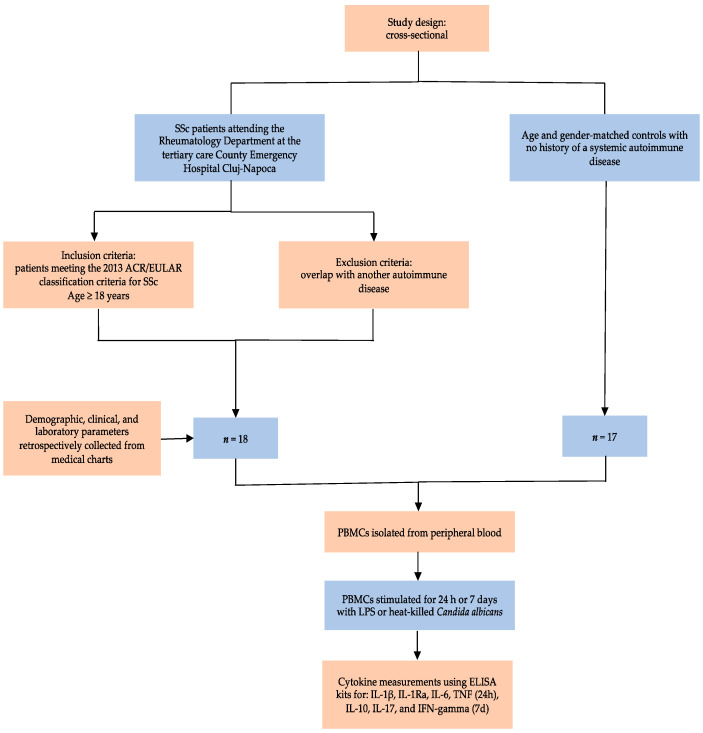
Diagram of the study design outlining inclusion and exclusion criteria, study participants, and variables.

A flow chart of the study methodology is depicted in Figure 4.

### 4.4. Statistical Analysis

Statistical analysis was performed using R software (4.2.3 version) with R Studio IDE (as an integrated development environment). Considering data distribution, statistical evaluation was conducted using the Student *t*-test or Wilcoxon rank-sum test when comparing 2 groups, and Spearman’s or Pearson’s correlation for measuring the association between 2 variables. A cutoff value for *p* < 0.05 was considered statistically significant. Box plots displaying median with quartiles were used to visualize the distribution and difference between controls and SSc patients. Base packages: xlsx, ggpubr, dplyr, ggplot2, stats. Running under Windows 10 x64, 22H2 (build 19045).

## 5. Conclusions

In summary, this study emphasized the role of cell-mediated immunity in the pathogenesis of SSc by demonstrating a generally elevated cytokine response in patients versus controls which is maintained throughout the course of the disease. The sustained PBMC reactivity seen in our patients up to 16 years after diagnosis supports the hypothesis that an innate immune memory could be present in SSc which enables patients to mount an increased inflammatory response to subsequent insults. Further exploration of these premises requires corroboration with genomic, transcriptomic, and metabolomic data. As previously mentioned, several limitations hindered our ability to establish clinically relevant correlations or define disease subtypes according to their cytokine signature. Validation of these preliminary findings and further mechanistic assessment in larger cohorts is warranted.

## Figures and Tables

**Table 1:** Baseline demographic, clinical, and laboratory disease parameters of SSc patients.

Characteristics	N	SSc, N = 18 ^1^
**Age** (years)	18	50 (45, 56)
**Gender**	18	
female		18 (100%)
**Disease duration** (years)	18	4.0 (2.2, 9.2)
**Disease duration** (early ≤3 years; late 3 years)	18	
early		7 (39%)
late		11 (61%)
**SSc subtype**	18	
diffuse cutaneous		9 (50%)
limited cutaneous		8 (44%)
sine scleroderma		1 (6%)
**Esophagitis**	15	6 (40%)
unknown		3
**ILD**	18	11 (61%)
**Arrhythmias requiring therapy**	18	1 (5.6%)
**Arthritis**	18	1 (5.6%)
**Digital ulcers**	18	
current		5 (28%)
never		8 (44%)
previous		5 (28%)
**Calcinosis**	18	4 (22%)
**Telangiectasia**	18	8 (44%)
**mRSS**	16	5.5 (4.0, 8.8)
unknown		2
**ANA**	18	
negative		1 (6%)
positive		17 (94%)
**Anti-Scl-70**	18	
negative		7 (39%)
positive		11 (61%)
**Anti-centromere**	6	
negative		1 (17%)
positive		5 (83%)
unknown		12
**Rheumatoid factor**	17	
negative		12 (70%)
positive		5 (30%)
unknown		1
**CRP** (mg/dL)	18	0.19 (0.14, 0.33)
**ESR** (mm/h)	18	9 (6,15)
**Uric acid** (mg/dL)	17	4 (3.28–5.1)
unknown		1
**FVC** (% predicted)	8	93 (88–96.5)
unknown		10
**DLCO** (% predicted)	6	66 (66–74)
unknown		12
**LVEF** (%)	13	62 (59–65)
unknown		5

^1^ median (IQR); n (%). SSc = systemic sclerosis; ILD = interstitial lung disease; mRSS = modified Rodnan skin score; ANA = antinuclear antibodies; CRP = C reactive protein; ESR = erythrocyte sedimentation rate; FVC = forced vital capacity; DLCO = diffusing capacity of carbon monoxide; LVEF = left ventricular ejection fraction; unknown = missing information.

## Data Availability

Data sharing is not applicable to this article.
